# Multifunctional Activity of *Syzygium aromaticum* Extracts Against *Candida albicans*: Free Radicals, Membrane Permeabilization and Cdr1p Localization

**DOI:** 10.3390/ijms26178571

**Published:** 2025-09-03

**Authors:** Daria Derkacz, Liliana Cebula, Anna Krasowska

**Affiliations:** Department of Biotransformation, Faculty of Biotechnology, University of Wroclaw, F. Joliot-Curie 14A, 50-383 Wroclaw, Poland; daria.derkacz@uwr.edu.pl (D.D.); liliana.cebula2@uwr.edu.pl (L.C.)

**Keywords:** *Candida albicans*, eugenol, plasma membrane

## Abstract

Eugenol is a compound with promising antimicrobial properties. The rising phenomenon of multidrug resistance of *Candida albicans* is driving researchers to search for new, alternative therapeutics that would synergize with conventional antifungal drugs. The aim of the present study is to investigate how eugenol and eugenol-based extracts impair *C. albicans* growth by generation of reactive oxygen species (ROS) and plasma membrane (PM) disruption. The methods that we applied involve structural analysis of eugenol extracts by HPLC, ATR-FTIR, and polyphenol detection. Additionally, determination of ROS level in *C. albicans* was performed using microscopic and flow cytometry studies and analysis of PM integrity (PI-staining, observation of PM transporter—Cdr1p—localization) and fluidity (fluorometric study). The results indicate that eugenol impacts fungal growth, and this corresponds with increased ROS levels and diminished PM fluidity in the *C. albicans* WT strain. *C. albicans* strains deprived of ergosterol (*erg11Δ/Δ*) exhibited lowered ROS level and no change in PM fluidity in response to the tested eugenol extracts, but they affected its growth and caused PM permeabilization and Cdr1p delocalization. These conclusions indicate that mode of action of eugenol can be related to disruption of PM structure by both ergosterol-dependent and -independent mechanisms. Ergosterol can play a crucial role in maintaining the PM integrity during treatment with lower concentrations of eugenol.

## 1. Introduction

Eugenol (C_10_H_12_O_2_; 4-allyl-2-methoxyphenol) is the major component of clove essential oils and extracts derived from *Eugenia caryophyllata*, also called *Syzygium aromaticum* [1,2]. Based on its structure, it is assigned to the group of phenol compounds. Concentrated extracts of eugenol have an oily consistency with a clear or yellow color. The important property of eugenol is its ability to scavenge free radicals and inhibit creation of reactive oxygen species (ROS) by receiving donated hydrogen atoms [3]. The antioxidant properties have been widely studied for scavenging 2,2-diphenyl-1-picrylhydrazyl (DPPH) free radical [4]. Moreover, this antioxidant activity is observed in low concentrations, while in higher doses, eugenol may promote the formation of free radicals and act as a prooxidant [3,5].

This natural compound demonstrates antimicrobial properties, including antifungal (e.g., *Candida* spp., *Penicillium* spp., *Aspergillus* spp., or *Fusarium* spp.) and antibacterial (e.g., *Escherichia coli* or *Pseudomonas aeruginosa*) activity [6,7,8,9]. Antimicrobial activity of eugenol was also investigated in combination with antibiotics. Synergistic action of eugenol and fluconazole combination has been proven against *C. albicans* [10]. Moreover, eugenol alone has no direct antimicrobial properties against tetracycline-resistant *Staphylococcus aureus*, but in combination with tetracycline, it potentiates the antibiotic action [11]. This proves that eugenol has a prominent potential in treatment of infections caused by resistant fungi and bacteria. Interestingly, there have been proposed new miconazole-based azoles derived from eugenol with promising antifungal activity (against *Candida* spp. and *Cryptococcus gatti*) [12].

In recent years, eugenol’s mode of action has been widely studied. The study involving *E. coli* showed that eugenol affected outer membrane of bacteria, causing leakage of intracellular content [8]. This supports the synergistic effect of eugenol with antibiotics—first, eugenol causes permeabilization of microbial plasma membrane (PM); second, antibiotics can effectively penetrate the microbial cells.

Antifungal properties of eugenol against *C. albicans* include an increase in intracellular reactive oxygen species (ROS), PM ATPase inhibition, or disruption of the biofilm structure [6]. Ahmad A. et al. proved that eugenol and its derivative (methyl eugenol) can inhibit sterol biosynthesis, leading to fungicidal effect in both susceptible and resistant *Candida* spp. [13]. This study shows that eugenol can exhibit similar mode of action as medically used antifungals (azoles), and the insight into the specific mechanism is essential to expand knowledge of new potential therapeutics. Additionally, analysis of the extracts derived from *Syzygium aromaticum* could be valuable because depending on the solvent used, clove extracts may contain other active components besides eugenol. Those bioactive substances are, e.g., eugenyl acetate, trans-caryophyllene, flavonoids, and hydroxycinnamic acids [14,15].

We believe that our research contributes to understanding the antifungal role of eugenol and eugenol-based extracts. During this research we involved a *C. albicans* strain deprived of ergosterol, which brings the new insight into the importance of sterol composition in eugenol’s interaction with PM and its role in inducing free radical production in fungal cells.

## 2. Results

### 2.1. Composition of Eugenol Extracts

#### 2.1.1. HPLC-UV Analysis

**Figure 1:**
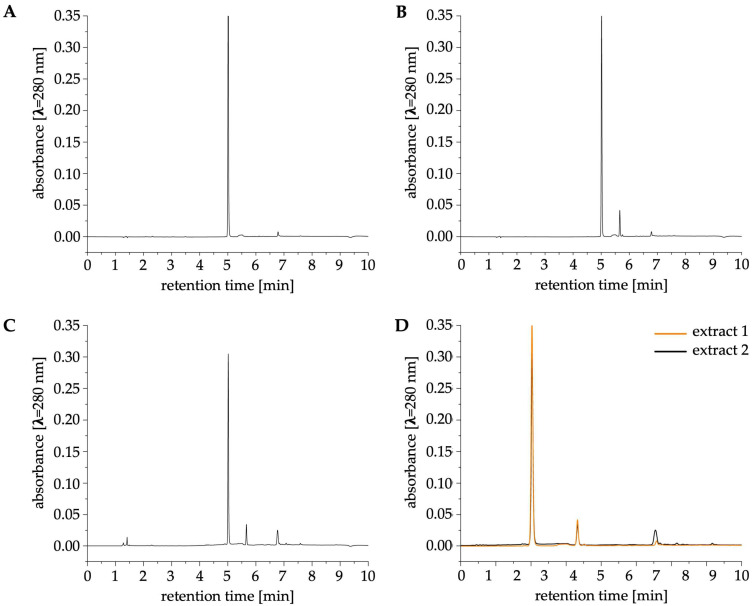
Chromatograms obtained after HPLC-UV analysis of a eugenol standard (0.78 mg/mL; (**A**)), extract 1 (**B**), and extract 2 (**C**), both diluted 1000 times. Merged chromatograms of the tested extracts are presented in (**D**). The detection was conducted using a UV lamp set to λ = 280 nm.

To determine composition of the tested eugenol extracts 1 (hexane-based extract) and 2 (concentrated extract), we performed an HPLC-UV (detection at λ = 280 nm) analysis (Figure 1). The content of eugenol in the analyzed extracts is presented in Table 1.

The HPLC-UV analysis revealed comparable absorption peaks at a retention time equal to 5 min for extracts 1 and 2 (Figure 1B–D). The retention time of the peak was consistent with that obtained for the eugenol standard (Figure 1A).

Based on the standard curve, the concentration of eugenol in extracts 1 and 2 was 662.71 and 553.18 mg/mL, respectively. Standard curve and chromatograms for different concentrations of eugenol standards are provided in Supplementary Materials (Figures S1 and S2).

#### 2.1.2. ATR-FTIR Analysis

**Figure 2:**
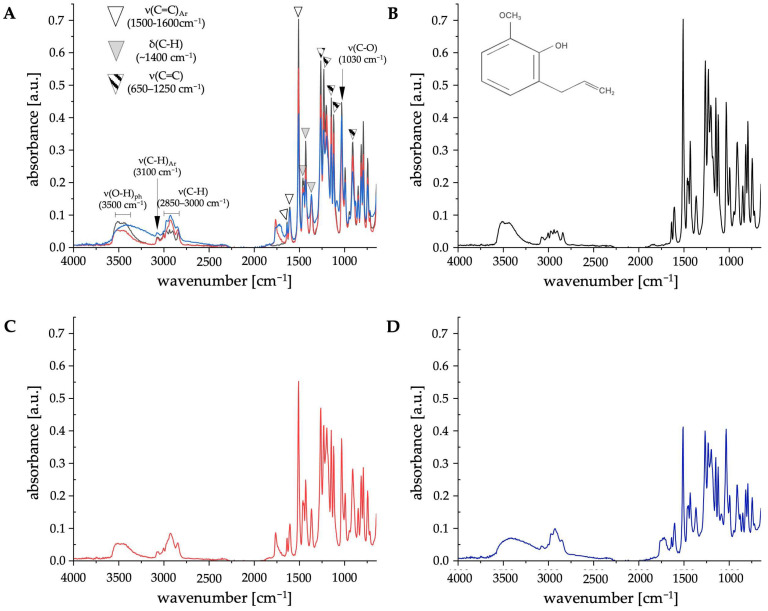
Merged spectra (**A**) of eugenol (**B**), extract 1 (**C**), and 2 (**D**) performed using ATR-FTIR (colors of lines represents eugenol, extract 1 and 2 as indicated in Figure 2B–D). In (**B**), the structural formula of eugenol is presented. The description of the bonds detected in specific wavenumber is indicated on merged spectra (**A**) and the bonds detected are following: ν(O-H)_ph_ (3500 cm^−1^)—stretching vibrations originating from the phenol –OH group; ν(C-H)_Ar_ (3100 cm^−1^)—stretching vibrations originating from the C-H bond of the aromatic group; ν(C-H) (2850–3000 cm^−1^)—stretching vibrations originating from the –CH_3_ and –CH_2_ groups; δ(C-H) (1370, 1450 cm^−1^)—deformation vibrations (bending) from −CH_3_ group; δ(C-H) (1465, 720 cm^−1^)—deformation vibrations (bending) from −CH_2_ group; ν(C=C) (650–1250 cm^−1^)—stretching vibrations from the C=C bond; ν(C=C)_Ar_ (1510, 1610, 1640 cm^−1^)—stretching vibrations from the C=C bond in aromatic group; ν(C-O) (1030 cm^−1^)—stretching vibrations from the C-O bond.

The HPLC-UV analysis revealed that the major component of the tested extracts was eugenol (Table 1). To confirm this, we performed an ATR-FTIR analysis of the extracts (Figure 2).

Presence of absorption peaks in the region 1500–1600 cm^−1^ in extracts 1 and 2 originates from stretching vibrations of aromatic C=C bonds, which are characteristic of eugenol structure (Figure 2B). Thus, ATR-FTIR confirmed that the major component of the tested extracts is eugenol. The different course of absorption peak is observed in the region of 3500 cm^−1^ in extract 2 (Figure 2D) compared to pure eugenol (Figure 2B) and extract 1 (Figure 2C). We assume that this is due to presence of ethanol in this concentrated extract, as this region reflects presence of ν(O-H) bonds. Additionally, ATR-FTIR data confirmed the HPLC-UV analysis that extract 2 contains a lower concentration of eugenol than extract 1 (Figure 2A).

### 2.2. Antifungal Activity of Eugenol Extracts Against Candida albicans

**Figure 3:**
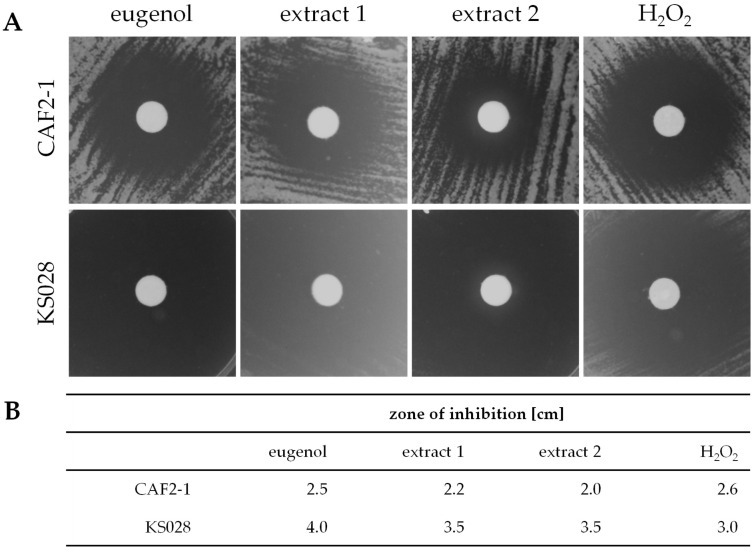
The growth inhibition zones (**A**) of *C. albicans* CAF2-1 (WT) and KS028 (*erg11Δ/Δ*) in the disk diffusion test. A total of 5 µL of concentrated eugenol, extract 1, extract 2, and hydrogen peroxide (H_2_O_2_; 450 mg/mL) were spotted on disks and then placed on YPD agar plates with cultures of *C. albicans* CAF2-1 and KS028 (starting OD_600_ equal to 0.2 and 0.4, respectively). Then, the YPD agar plates were incubated for 24 h at a temperature of 28 °C, and then photographs were taken. The zones of inhibition (**B**) were measured as the diameter [cm].

To analyze the antifungal activity of eugenol extracts 1 and 2, the disk diffusion test was employed (Figure 3A). H_2_O_2_ was used as an oxidizing compound, serving as a standard in the research on ROS detection in the presence of eugenol and extracts, as shown in later sections of this work. Growth inhibition zones for *C. albicans* CAF2-1 (WT) and KS028 (*erg11Δ/Δ*) were measured, and data are presented in a table in Figure 3B.

All the tested compounds resulted in growth inhibition of both *C. albicans* CAF2-1 (WT) and KS028 (*erg11Δ/Δ)*. For *C. albicans* CAF2-1 the largest zones of inhibition were detected in presence of eugenol (2.5 cm) and hydrogen peroxide (H_2_O_2_; 2.6 cm). In case of *C. albicans* KS028, the strongest growth inhibition was observed for eugenol (4 cm), and then for both the tested extracts (3.5 cm). *C. albicans* KS028 is more susceptible to all the tested compounds compared to *C. albicans* CAF2-1.

**Figure 4:**
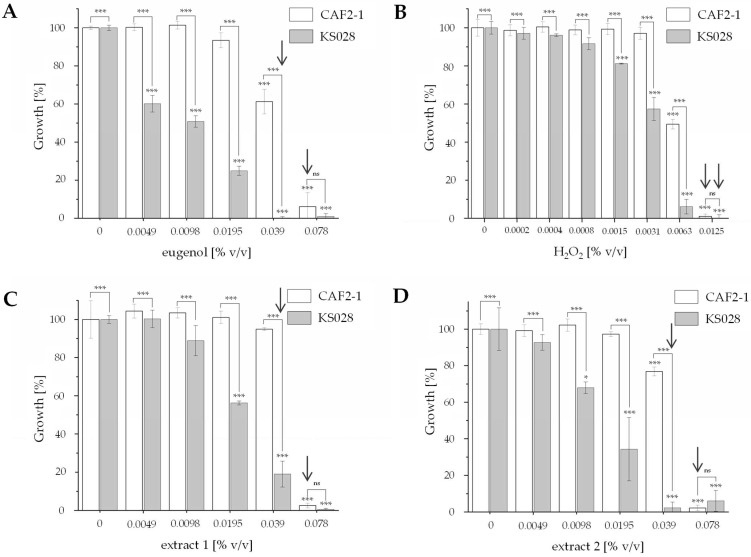
Growth of *C. albicans* CAF2-1 (WT) and KS028 (*erg11Δ/Δ*) in presence of eugenol (**A**), extract 1 (**B**), extract 2 (**C**), and hydrogen peroxide (H_2_O_2_; (**D**)). The concentration indicated for extracts 1 and 2 represents the concentration of eugenol in specific dilutions of testes extracts (the dilution of both extracts was performed in range of 0.078–0.0049% *v*/*v*). *C. albicans* CAF2-1 and KS028 were cultured in YPD medium supplemented (or not) with the tested compounds on 96-well plates for 24 h (28 °C, stationary). Then the optical density (OD) at λ = 600 nm was measured, and the growth (%) was calculated comparing to OD_600_ for *C. albicans* cultured without presence of any compound (control conditions). Gray arrows indicate MIC50 for the tested *C. albicans* strains. The experiment was performed in 3 biological repetitions (±SD, ns ≥ 0.05; * *p* < 0.05; *** *p* < 0.001).

Subsequently, determination of MIC50 (minimal inhibitory concentration that causes 50% *C. albicans* growth inhibition) of eugenol, H_2_O_2_, and extracts 1 and 2 was performed. The obtained data is presented in Figure 4.

*C. albicans* KS028 is more susceptible to all the tested compounds than the CAF2-1 strain (Figure 4). The MIC50 for *C. albicans* CAF2-1 for eugenol, H_2_O_2_, and extracts 1 and 2 were 0.078, 0.0125, 0.078, and 0.078% *v*/*v*. The corresponding MIC_50_ concentrations of eugenol in eugenol, extract 1, and extract 2 were 825.3, 516.9, and 431.5 µg/mL, respectively, as quantified using the HPLC-UV method. The MIC50 values for the tested extracts were lower when we consider the eugenol content in the extracts compared to pure eugenol, which indicates greater antifungal potential of those extracts, apart from the fact that eugenol is the major component of those extracts (Figures S1 and S2, Supplementary Materials).

For *C. albicans* KS028, the MIC50 of eugenol, H_2_O_2_, and extracts 1 and 2 values were 0.0195, 0.0063, 0.039, and 0.039% *v*/*v* (for eugenol and extracts 1 and 2, the MIC50 concentration of eugenol was 206.3, 258.5, and 215.7 µg/mL, respectively).

### 2.3. Oxidative Stress Induced in Response to Eugenol Extracts

#### 2.3.1. DPPH Free Radical Scavenging

To determine the antioxidant potential of eugenol and the tested extracts, the scavenging of DPPH free radical was measured. Considering that our research involves a *C. albicans* strain deprived of ergosterol from PM, the antioxidant potential of ergosterol was also measured. Data are presented in Table 2.

The level of DPPH free radical scavenging was dependent on concentration of all the tested compounds. Pure eugenol exhibited the strongest antioxidant properties at t = 0 min in comparison to extracts 1 and 2, but after 10 min, the A_517_ was comparable. Eugenol and the tested extract exhibited similar antioxidant properties as trolox (positive control) in t = 10, 60, and 90 min, while ergosterol exhibited moderate DPPH free radical scavenging compared to trolox positive control, eugenol, and extracts.

#### 2.3.2. Cellular ROS Level

**Figure 5:**
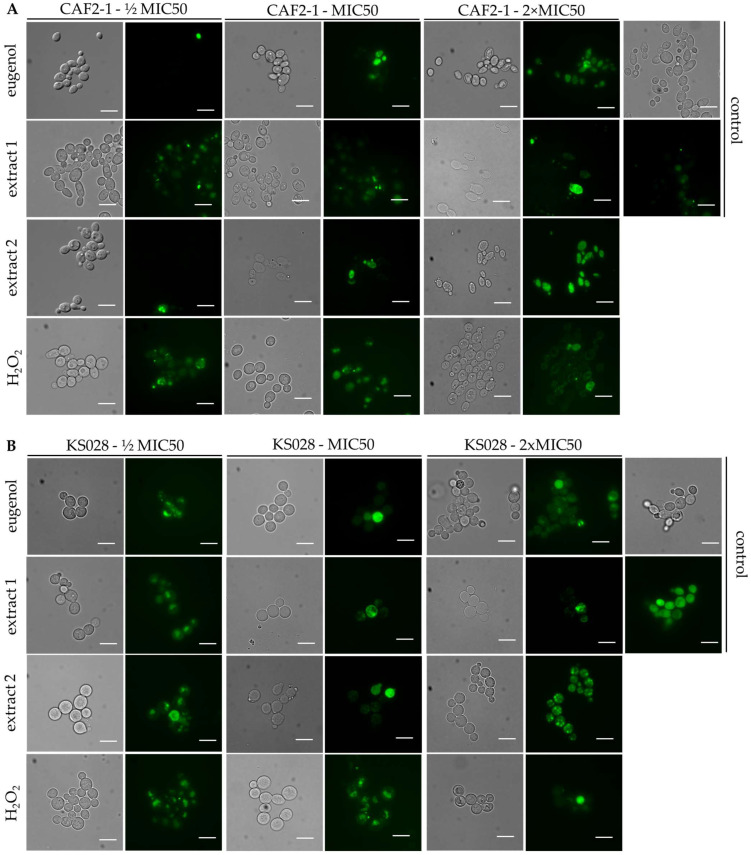
Representative microphotographs of ROS generated in *C. albicans* CAF2-1 (WT) (**A**) or KS028 (*erg11Δ/Δ*) (**B**) cells visualized with the DCFDA probe. The cells were treated with different concentrations (½xMIC50, MIC50, and 2xMIC50) of eugenol, extract 1, extract 2, or H_2_O_2_. The MIC50 values were, respectively, 0.078, 0.078, 0.078, and 0.0125% *v*/*v* for the CAF2-1 strain and 0.0195, 0.039, 0.039, and 0.0063% *v*/*v* for the KS028 strain. Scale bar = 10 µm.

**Figure 6:**
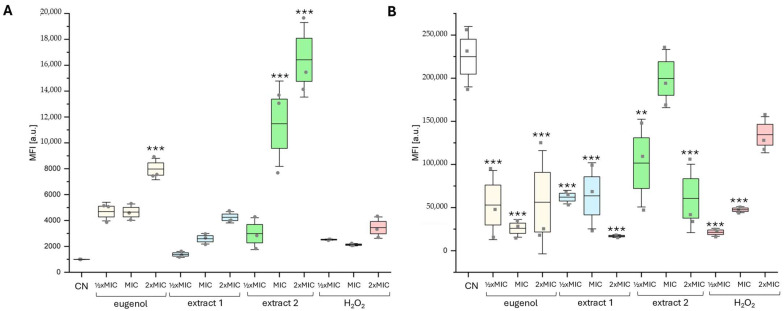
Flow cytometry analysis of ROS generated in *C. albicans* CAF2-1 (WT) (**A**) and KS028 (*erg11Δ/Δ*) (**B**) strains cultured in YPD medium for 4 h alone (negative control; CN) or in presence of eugenol, extract 1, extract 2, and H_2_O_2_ (concentrations ½xMIC50, MIC50, and 2xMIC50). The MIC50 values were, respectively, CAF2-1—0.078, 0.078, 0.078, and 0.0125% *v*/*v*; KS028—0.0195, 0.039, 0.039, and 0.0063% *v*/*v*. The experiment was performed in 3 biological repetitions (±SD; ** *p* < 0.01; *** *p* < 0.001).

The influence of the tested compounds on reactive oxygen species (ROS) production in *C. albicans* cells was determined using a DCFDA probe and analyzed using fluorescence microscopy (Figure 5) and flow cytometry (Figure 6).

The microscopic analysis revealed that in control conditions, *C. albicans* KS028 exhibits stronger fluorescence than the WT strain. Treatment of *C. albicans* CAF2-1 with eugenol, extracts 1 and 2, and H_2_O_2_ led to a concentration-dependent increase in DCFDA fluorescence (½xMIC50, MIC50, and 2xMIC50). Such a trend was not observed for the KS028 strain, with DCFDA fluorescence displaying irregular changes across different concentrations. This particularly applies to the ½xMIC50 concentration of all the tested compounds.

The flow cytometry analysis confirmed the microscopic data that *C. albicans* KS028 exhibits significantly higher ROS levels in control conditions than the CAF2-1 strain (CN in Figure 6A,B). In the *C. albicans* CAF2-1 strain, the higher ROS level correlates with increasing concentration of the tested compounds. Such a tendency is not present in case of the KS028 strain, where fluorescence is significantly reduced in all the tested conditions compared to CN.

### 2.4. Candida albicans PM Integrity Maintenance in Response to Eugenol Extracts

#### 2.4.1. Permeabilization of Fungal PM

**Figure 7:**
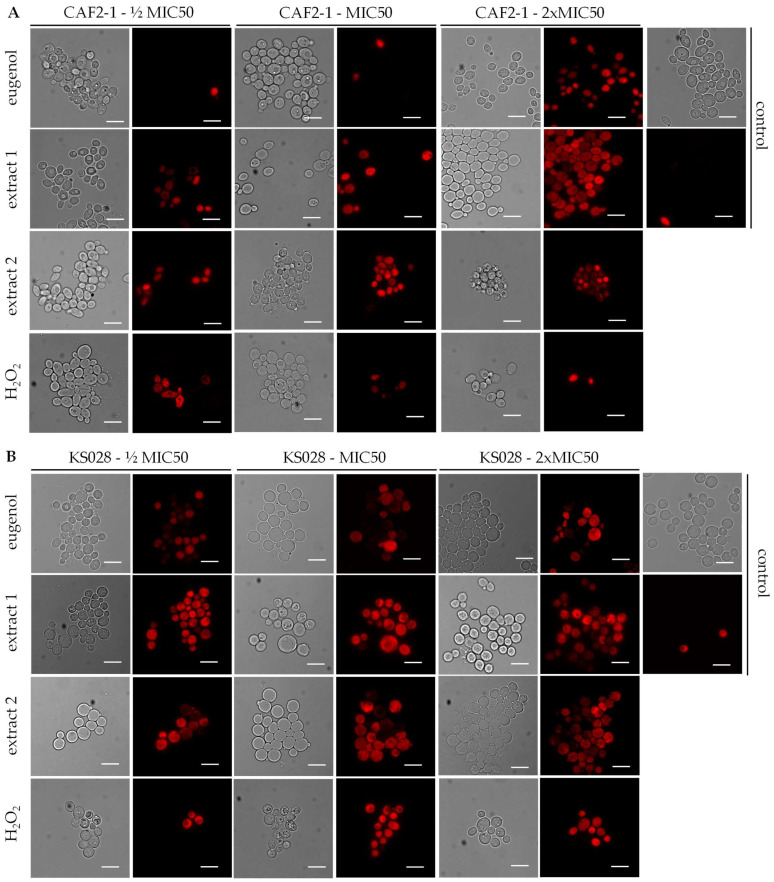
Representative microphotographs of propidium iodide (PI)-stained *C. albicans* CAF2-1 (WT) (**A**) or KS028 (*erg11Δ/Δ*) (**B**) cells after treatment with different concentrations (½xMIC50, MIC50, and 2xMIC50) of eugenol, extract 1, extract 2, or H_2_O_2_. The MIC50 values were, respectively, 0.078, 0.078, 0.078, and 0.0125% *v*/*v* for the CAF2-1 strain and 0.0195, 0.039, 0.039, and 0.0063% *v*/*v* for the KS028 strain. Scale bar = 10 µm.

To test if the eugenol extracts alter the PM permeabilization, the PI analysis was performed (Figure 7 and Table 3).

For the CAF2-1 (WT) strain, eugenol treatment resulted in a significant increase in PI fluorescence at concentrations of 2xMIC50 compared to the control conditions. In contrast, presence of lower concentrations (½xMIC50 or MIC50) of extracts 1 and 2 resulted in a higher percent of PM permeabilization (for ½xMIC50 41.13 and 35.32%; for MIC50 45.67 and 35.67%, respectively) compared to ½xMIC50 and MIC50 concentrations of eugenol (for ½xMIC50 and MIC50 8.79 and 9.76%, respectively). Moreover, the disruption of cells after treatment with 2xMIC50 of extracts 1 and 2 appears to be stronger than for the same concentration of pure eugenol (Table 3).

The *C. albicans* KS028 (*erg11Δ/Δ*) strain is more sensitive to all the tested compounds in lower concentrations than the WT strain, and the permeabilization in ½xMIC50 is higher than 50% in all the cases except H_2_O_2_ (Table 3). Additionally, a significant difference in permeabilization was detected between the tested strains in almost all the analyzed conditions. This suggests that ergosterol plays a crucial role in maintaining the integrity of *C. albicans* PM during eugenol and eugenol-based extract treatment.

#### 2.4.2. Fluidity of Fungal PM

To analyze if eugenol and extracts 1 and 2 have an impact on PM fluidity depending on the presence (WT strain) or absence (KS028 strain) of ergosterol, the fluorometric study of laurdan probe incorporated to *C. albicans* PM was applied, and the results are shown in Table 4.

Treatment of *C. albicans* CAF2-1 with eugenol, extracts 1 and 2, and H_2_O_2_ contributed to reduced PM fluidity, expressed as decreased GP value. *C. albicans* KS028 exhibited decreased PM fluidity compared to the WT strain due to absence of ergosterol, but KS028 (*erg11Δ/Δ*) treatment with the tested compound did not significantly change the GP value.

#### 2.4.3. PM Localization of CaCdr1p

**Figure 8:**
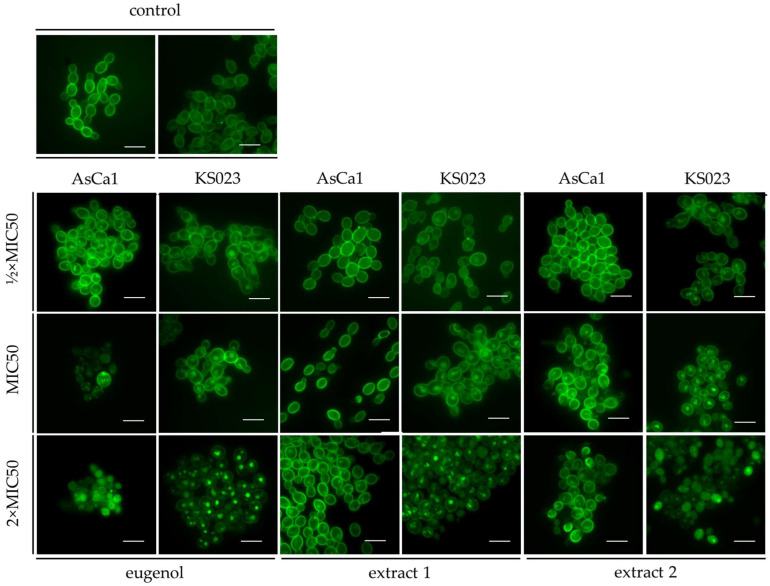
The localization of Cdr1p-GFP after treatment of *C. albicans* AsCa1 (same as CAF2-1 but with Cdr1p-GFP) and KS023 (same as KS028 but with Cdr1p-GFP) with eugenol and extracts 1 and 2. *C. albicans* AsCa1 and KS023 were cultured for 8 h (20 mL of YPD medium, 28 °C, 120 rpm) with (or without—control) addition of ½xMIC50, MIC50, and 2xMIC50 of eugenol, extract 1, and extract 2 (MIC50 values (%*v*/*v*) for AsCa1 were, respectively, 0.078, 0.078, and 0.078 and for KS023: 0.0195, 0.039, and 0.039). After this the microscopic observation was performed (scale bar = 10 µm).

Delocalization of PM proteins (e.g., Cdr1p) is another indicator of disruption of PM structure and integrity. Therefore, we applied a microscopic study of Cdr1p-GFP localization in PM in response to different concentrations of eugenol and eugenol-based extracts. The data are presented in Figure 8. To determine if those *C. albicans* AsCa1 and KS023 (Cdr1p-GFP) strains exhibit the same growth inhibition, we performed viability tests in the same manner as for the CAF2-1 and KS028 strains (Figure S3).

Interestingly, the complete Cdr1p delocalization from PM was observed in *C. albicans* WT at the highest concentration of eugenol, while only partial delocalization was detected after extract 1 and 2 treatment. The effect of eugenol was also the most spectacular among all the tested compounds for *C. albicans* KS023 in 2xMIC50 concentration, while in MIC50, a similar effect of all the tested compounds was observed (partial Cdr1p delocalization).

## 3. Discussion

The main phenolic component of clove essential oil, eugenol, exhibits promising antibacterial and anti-inflammatory properties [16]. Therefore, it is important to investigate eugenol’s mode of action against pathogenic fungi, e.g., *C. albicans*. Here, we confirmed that hexane-based (extract 1) and concentrated (extract 2) extracts from *Syzygium aromaticum* have an impact on *C. albicans* CAF2-1 (WT) and KS028 (*erg11Δ/Δ*) strains’ growth (Figure 3 and Figure 4). The HPLC-UV and ATR-FTIR analysis confirmed that the major component of both the tested extracts is eugenol (Figure 1 and Figure 2). Nevertheless, we observed slight differences in antifungal activity of extracts 1 (for CAF2-1, MIC50 = 516.9 µg/mL of eugenol content) and 2 (for CAF2-1, MIC50 = 431.5 µg/mL of eugenol content) based on a growth assay (Figure 4). Both extracts exhibited higher potential to inhibit the *C. albicans* growth compared to pure eugenol standard (for CAF2-1, MIC50 = 825.3 µg/mL) in terms of eugenol content. It is worth mentioning that ATR-FTIR analysis revealed an additional band at 1760 cm^−1^, which corresponds with presence of eugenol acetate (Figure 2) [17]. Also, the slight differences in bands present at 3200–3700 cm^−1^ (especially in extract 2) were observed for the tested extracts. We assume that this is due to presence of ethanol in this concentrated extract 2. Given that this region reflects presence of ν(O-H) bonds, other phenolic compounds could be responsible for this shift (e.g., flavonoids like quercetin and kaempferol or other phenolic acids, e.g., ferulic, caffeic, or salicylic acids) [18]. This is also supported by the polyphenol analysis, which resulted in similar (extract 1) or elevated levels (extract 2) of polyphenols compared to eugenol (Figure S4). Since eugenol is a major part of extracts 1 and 2, this leads us to the conclusion that there are other compounds with polyphenolic properties that can act synergistically with eugenol on *C. albicans* growth inhibition. The MIC50 concentration for eugenol and the analyzed extracts is consistent with data obtained by different research groups, and this range of concentrations also applies to *C. albicans* isolates [19,20]. The disk diffusion test revealed that extract 1 caused a larger *C. albicans* WT growth inhibition zone compared to extract 2 (⌀ = 2.0 vs. 2.2 cm, respectively), but still smaller than pure eugenol (⌀ = 2.5 cm; Figure 3). This can be associated with a lower concentration of eugenol in extract 2 (553.18 μg/mL) in comparison to extract 1 (662.71 μg/mL; Table 1). For *C. albicans* KS028, for extracts 1 and 2 growth inhibition zone was equal to ⌀ = 3.5 cm (⌀ = 4 cm for pure eugenol). This could be due to the fact that the KS028 strain is more sensitive to presence of environmental stressors, and it exhibits an impaired growth rate compared to the CAF2-1 (WT) strain.

Eugenol was reported to exhibit antioxidant properties [16]. In our study the ½xMIC50 and MIC50 concentrations of eugenol (for CAF2-1 (WT) and KS028 (*erg11Δ/Δ*), respectively) initially (t = 0) resulted in lower ability for scavenging DPPH free radical compared to the trolox positive control (Table 2). This difference was compensated after 10 min of incubation, where percentage of DDPH scavenging was even with that obtained for the positive control. Similar observation was made for extracts 1 and 2, except the initial percent of DDPH scavenging was even lower compared to pure eugenol (22.96 and 21.18 vs. 79.22%, respectively). Ergosterol exhibited no antioxidant properties at t = 0 or 10 min, where the percent of DPPH scavenging was comparable to the negative control (H_2_O_2_), but prolonged incubation resulted in significantly increased rate of DPPH free radical scavenging.

Treatment of *C. albicans* CAF2-1 with H_2_O_2_ (positive control) in MIC50 (0.0125% *v*/*v*) and 2xMIC50 concentrations resulted in greater fluorescence of the DCFDA probe compared to the control conditions (Figure 5 and Figure 6A). The increasing concentration of eugenol and extracts 1 and 2 resulted in higher levels of generated ROS in the CAF2-1 cells. A similar observation, that increasing doses of eugenol result in elevated levels of ROS, was noted in Shahina Z. et al.’s work, but the range of used concentrations in this study was different [19].

An interesting observation was noted in case of the *C. albicans* KS028 strain (Figure 4B). In the control conditions, the KS028 (*erg11Δ/Δ*) strain exhibited a high ROS level (MFI of the DCFDA probe for the CAF2-1 and KS028 strains was 1000 and 225,000 a.u., respectively). This could be because enzymes of the late ergosterol biosynthesis pathway (including Erg11p, which *C. albicans* KS028 lacks) are involved in utilizing iron as a cofactor [21]. Therefore, absence of Erg11p leads not only to production of toxic methylated forms of sterols but also results in accumulation of iron. This iron excess can translate into ROS generation inside cells through Fenton or Haber–Weiss reactions [22]. Surprisingly, treatment of the KS028 strain with eugenol, tested extracts, and H_2_O_2_ resulted in reduction in ROS levels inside cells. According to Table 2 ergosterol has a slight potential for scavenging DPPH free radicals in vitro. The same (but to a greater extent) was observed for eugenol and extracts 1 and 2. This proves that ergosterol plays a crucial role in maintaining physiological ROS levels, and its absence correlates with changed protective mechanisms of eugenol action in the KS028 (*erg11Δ/Δ*) strain.

The *C. albicans* cells’ PI staining revealed the dose-dependent permeabilization of fungal PM (Figure 7). According to Table 3, the percentage of permeabilized CAF2-1 (WT) cells was higher for extracts 1 and 2 than for pure eugenol. This indicates that those extracts possess some additional components that make those extracts more damaging to PM. *C. albicans* KS028 (*erg11Δ/Δ*) exhibits a greater percentage of permeabilized PM compared to the CAF2-1 (WT) strain in almost all the tested conditions, probably due to growth impairment and abnormal construction of PM according to absence of ergosterol. Pinto et al. showed, using the same PI staining technique, that even after short incubation (15 min) of *C. albicans* cells, clove oil and eugenol resulted in significantly increased PM permeabilization [23]. Taking this data together, the mode of action of eugenol is directly focused on disruption of membrane integrity (including fungal and bacterial membranes) [7,24].

Moreover, in work of Pinto et al., they proved that after eugenol treatment, ergosterol level in *C. albicans* decreases, and the same trend was observed for azole-resistant *C. krusei* [23]. Our analysis also confirmed significant reduction of ergosterol level in *C. albicans* CAF2-1 after exposure of cells to eugenol, extract 1 and 2 (Table S1). This correlates with our finding that for the *C. albicans* WT strain, the fluidity of PM decreases after eugenol and extracts 1 and 2 treatment (Table 4). As we previously reported, the GP value was significantly lower for the KS028 strain compared to WT [25]. Interestingly, after treatment with different concentrations of eugenol and extracts, the general polarization (GP) of laurdan probe for *C. albicans* WT strain is like that observed for the *erg11Δ/Δ* strain.

Treatment of the KS028 (*erg11Δ/Δ*) strain with different concentrations of eugenol and extracts did not significantly change fluidity of PM. These data suggest that one of the possible eugenol modes of action is disrupting integrity of PM by altering the ergosterol level. Considering other data obtained in the present study (growth inhibition tests—Figure 1 and Figure 2; increased PM permeabilization—Figure 7 and Table 3), eugenol exhibits fungicidal activity even in absence of ergosterol in PM. This hypothesis is also supported by analysis of Cdr1p-GFP localization. *C. albicans* KS023 (*erg11Δ/Δ*; *CDR1*-GFP) showed delocalization of Cdr1p-GFP in lower concentrations of eugenol and the tested extracts (Figure 8), while *C. albicans* AsCa1 (WT; *CDR1*-GFP) exhibited partial retention of this protein in PM after treatment with highest concentrations of the tested compounds. Therefore, we believe that obtaining this data for *C. albicans* deprived of ergosterol from PM will contribute to expanding knowledge on eugenol’s mechanism of action.

Eugenol-rich clove extracts can be considered valuable therapeutics for the treatment of *Candida* spp. infections, considering that eugenol demonstrated antifungal effects with its potential for clinical application in topical treatment of mucosal and skin candidiasis [26]. It has been reported that eugenol synergistically enhances the activity of antifungal drugs, such as fluconazole, by restoring susceptibility in previously resistant strains of *C. albicans* [10] and by inhibiting 14α-demethylase, which results in decreased ergosterol content in the fungal cells [27,28]. Besides its antifungal activity, eugenol also possesses other promising properties, such as antihypercholesterolemic effects through the inhibition of the squalene epoxidase enzyme, which is present in both human and fungal cells. This dual action—targeting both cholesterol regulation and fungal viability—highlights its value in therapeutic contexts [28]. This antifungal effect of eugenol corresponds with activity of other plant-derived compounds, e.g., thymol, a major constituent of thyme oil, which also inhibits ergosterol biosynthesis and disrupts the integrity of the fungal plasma membrane [29]. Regarding eugenol’s clinical applications, the World Health Organization has classified it as generally recognized as safe (GRAS), but it is worth mentioning that used in high concentration can cause side effects, e.g., localized irritation of the skin or allergic contact dermatitis [30]. Nevertheless, further analysis of clove extracts’ antifungal potential could translate into the creation of a new strategy for combating candidiasis in the future.

## 4. Materials and Methods

### 4.1. Experimental Design

**Figure 9:**
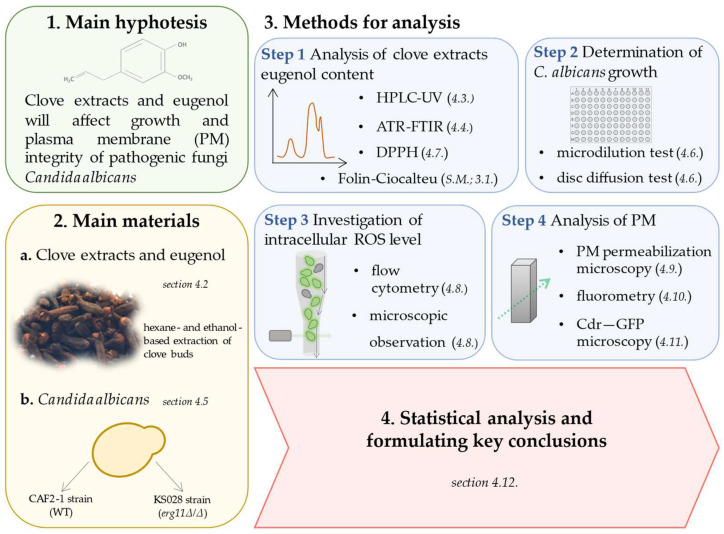
Graphical presentation of experimental design. The reference is to the Materials and Methods Section, where the detailed methodology is written in italics and in brackets (S.M.—Supplementary Materials). The figure was self-made and prepared using the PowerPoint program.

Experiments were designed to investigate the influence of clove extracts on *C. albicans* growth and plasma membrane integrity. The procedures utilized during this study are illustrated in Figure 9.

All detailed information about methodology is provided in dedicated subsections of Materials and Methods.

### 4.2. Chemicals

Eugenol extracts from *Syzygium aromaticum* were purchased in Green Zebras (Wroclaw, Poland). In case of both extracts, the plant material (dried flower buds) was crushed and then subjected to extraction with the following solvents: hexane (extract 1) and 96% *v*/*v* ethanol (extract 2, maximally concentrated to 98%). The ratio of plant mass to raw material was 1:4. Extraction with 96% *v*/*v* ethanol and hexane was carried out using the maceration technique with stirring for 30 min. The obtained extracts were filtered and then concentrated using a rotary vacuum evaporator.

Eugenol, laurdan, DPPH, propidium iodide, and DCFDA were purchased from Thermo Fischer Scientific (Waltham, MA, USA). Other materials were purchased as follows: NaCl (StanLab, Lublin, Poland); yeast extract and peptone (Bacto, Thermo Fischer Scientific, Waltham, MA, USA); glucose and agar (manufacturer: Bioshop; distributor: EPRO; Warsaw, Poland); PBS tablets, H_2_O_2_ 30% solution, and trolox (Merck, Darmstadt, Germany); methanol LC/MS grade (Chempure, Westland, MI, USA).

### 4.3. HPLC-UV Analysis of Eugenol Extracts

Analysis was performed based on the Inam F. et al. protocol [31] with modifications. Eugenol standard was serially diluted in methanol (LC/MS grade) from the original stock solution (c = 1.067 g/mL) to achieve eugenol concentrations ranging from 6.250 to 0.195 mg/mL (standard curve). The extracts were diluted 1000 times in methanol.

The HPLC analysis was performed using KINETEX^®^ 5 μm EVO C18 (100 Å, 150 mm × 4.6 mm) column and HPLC-UV system (AcquityArc, Waters, Milford, MA, USA). The column temperature was set to 40 °C, and the samples were kept at 21 °C. The mobile phase was acetonitrile and water, both with addition of 0.1% formic acid. The separation method is presented in Table 5.

The flow rate was 1.0 mL/min. The quantitative detection was conducted with a UV lamp set to 280 nm, and quantification of eugenol content in the extracts was calculated based on the standard curve for eugenol standard.

### 4.4. ATR-FTIR Analysis of Eugenol and Extracts

Analysis of eugenol and extract 1 and 2 compositions was based on Fourier-transform infrared spectroscopy (FTIR) using a Cary 630 FTIR spectrometer (Agilent Technologies, Santa Clara, CA, USA) equipped with a diamond crystal adapter for attenuated total reflection spectroscopy (ATR; Agilent Technologies). The solutions of eugenol and the tested extracts (5 µL) were placed and dried on a diamond crystal, and then the spectra were recorded in range of 750–4000 cm^−1^.

### 4.5. Strains and Culture Conditions

The *C. albicans* strains used in this study are listed in Table 6.

The *C. albicans* CAF2-1 strain was a kind gift from Prof. D. Sanglard (Lausanne, Switzerland).

The *C. albicans* strains were routinely grown in YPD (1% yeast extract, 1% peptone, 2% glucose, and agar in a final concentration of 2% was used for medium solidification) for 24 h, at a temperature of 28 °C, in a shaking incubator (120 rpm) if not indicated otherwise.

### 4.6. Susceptibility Testing and Determination of Minimal Inhibitory Concentrations

Susceptibility testing was performed using the disk diffusion method according to the CLSI M44-A2 guidelines with slight changes [34]. The overnight cultures of the *C. albicans* strains in YPD medium were washed three times with 0.9% saline solution (0.9% NaCl in H_2_O_dd_; 3 min, 7500 rpm) and resuspended in 0.9% saline solution. The optical density (OD, λ = 600 nm) of the cell suspensions was then adjusted to 0.2 (for CAF2-1 and AsCa1) or 0.4 (for KS023 and KS028) and spread on YPD agar plates. Then, disks with the tested compounds were placed on agar (5 µL per disk of concentrated extracts, eugenol, or H_2_O_2_) and then incubated for 24 h, at a temperature of 28 °C, stationary. After this, the plates were photographed using a FastGene^®^ B/G GelPic imaging box (Nippon Genetics; distributor: Abo, Gdańsk, Poland), and the size of the growth inhibition zone was measured.

Determination of minimal inhibitory concentration, which resulted in 50% growth inhibition (MIC50) for tested compounds, was performed as described before, according to the CLSI M27-A3 guidelines with previously reported changes [35,36,37]. The tested compounds were prepared as serial dilutions in YPD medium (0.0049–0.078% *v*/*v* for eugenol, extract 1, and extract 2; 0.000195–0.2% *v*/*v* for H_2_O_2_) in 96-well culture plates (Sarstedt; final volume = 50 µL per well). Then, overnight cultures of the *C. albicans* strains in YPD medium were adjusted to OD_600_ = 0.02, and 50 µL of the cultures was added to the wells containing diluted compounds (final volume = 100 µL). The plates were incubated for 24 h, at a temperature of 28 °C, in a stationary incubator, and then the OD_600_ of the cultures was recorded using Asys UVM 340 (Biogenet, Józefów, Poland). The growth inhibition for the tested strains was expressed as a percentage of growth according to the negative control (*C. albicans* cultures without the tested compounds in YPD medium). The experiment was performed in three independent repetitions.

### 4.7. Analysis of DPPH Free Radical Scavenging Potential

Determination of antioxidant activity of tested compounds was assessed with the 1,1-diphenyl-2-picrylhydrazyl (DPPH) free radical assay. DPPH radical solution (7.1 × 10^−5^ M) in methanol was freshly prepared prior to the experiment. In a 96-well plate, 20 μL of extracts, eugenol, and ergosterol (all dissolved in methanol; concentration of eugenol and extracts 1 and 2 was equal to 0.039% *v*/*v*; concertation of ergosterol, trolox, and H_2_O_2_ was 400, 500 μg/mL, and 0.0125% *v*/*v*, respectively) were added to the wells, followed by the addition of 180 μL of DPPH solution. Subsequently, the plate was incubated for 90 min (37 °C, 200 rpm), and at specific time points (0, 10, 60, and 90 min), absorbance at λ = 517 nm was recorded using the Spark multimode microplate reader (Tecan, Männedorf, Switzerland). To determine the scavenging activity of compounds against DPPH radicals, the reduction in absorbance at 517 nm was monitored (with methanol absorbance subtracted as background), and the percentage inhibition was calculated according to the control (DPPH alone). All the measurements were performed in three independent replicates.

### 4.8. Analysis of Intracellular ROS Production

Assay was performed with usage of 2′,7′-dichlorodihydrofluorescein diacetate (H_2_DCFDA probe) as described by Shahina Z. et al. [19], with modifications. After 24 h of culture, suspensions of *C. albicans* cells were prepared (YPD, OD_600_ = 0.2 in 5 mL) and then incubated for 4 h (28 °C, 120 rpm) with eugenol, extract 1, extract 2, or H_2_O_2_ as a positive control at concentrations: ½MIC50, MIC50, and 2xMIC50. MIC50 values for CAF2-1 were, respectively, 0.078, 0.078, 0.078, and 0.0125% *v*/*v*, and for KS028—0.0195, 0.039, 0.039, and 0.0063% *v*/*v*. The cells were then harvested (4500 rpm, 5 min), washed twice with PBS, and resuspended in PBS with the H_2_DCFDA probe (10 µM). After 30 min of incubation (30 °C, stationary), the cells were harvested (4500 rpm, 5 min), washed twice with PBS, and suspended in 1 mL PBS. Subsequently, a subset of the stained cells was concentrated and observed under a microscope equipped with a Zeiss Axiocam 503 mono microscope camera and a Zeiss HBO100 mercury lamp (Poznań, Poland), while the remaining cells were analyzed using flow cytometry. Quantification of the fluorescence signal of oxidized the DCFDA probe, corresponding directly to intracellular ROS levels, was performed within the FITC channel (Ex/Em = 488/519 nm) as mean fluorescent intensity (MFI). For each repetition, 20,000 events were collected using a NovoCyte 2060R flow cytometer (Agilent Technologies, Santa Clara, CA, USA). Signals from the cells not treated with the probe (background) were measured and subtracted from the fluorescence of the treated samples.

### 4.9. Analysis of Membrane Permeability

Plasma membrane permeability was assessed using propidium iodide (PI) cell staining according to a previously described protocol [38] with modifications. Briefly, *C. albicans* cells from 24 h of culture were resuspended in YPD (OD_600_ = 0.2 in 5 mL) and then exposed to the tested compounds for 4 h (28 °C, 120 rpm) to eugenol, extract 1, extract 2, and H_2_O_2_ at ½MIC50, MIC50, and 2xMIC50 (concentrations at the MIC50 values were, respectively, 0.078, 0.078, 0.078, and 0.0125% *v*/*v* for CAF2-1 and 0.0195, 0.039, 0.039, and 0.0063% *v*/*v* for KS028. The cells were then harvested (4500 rpm, 5 min), washed twice with PBS, and then adjusted to OD_600_ = 0.3 in 1 mL PBS. The cells were stained with propidium iodide in a final concentration of 4 μg/mL (RT, 5 min, in the dark). After staining, the cells were washed twice with PBS (4500 rpm, 5 min), concentrated, and observed under a Zeiss Axio Imager A2 microscope equipped with a Zeiss Axiocam 503 mono microscope camera and a Zeiss HBO100 mercury lamp (Poznań, Poland). The percentage of plasma membrane permeabilization was assessed by counting the PI-positive cells out of at least 100 cells in three independent repetitions for each condition.

### 4.10. Analysis of Membrane Fluidity

The assay was based on a previously described method [39] and according to our method [23]. Suspension of *C. albicans* was prepared as indicated for the permeabilization assay (YPD, OD_600_ = 0.2 in 5 mL, 4 h of culture). Then the cells were harvested (4500 rpm, 5 min) and washed three times with PBS. Then, cell suspensions (OD_600_ = 0.1, 3 mL in PBS) were incubated with laurdan (final conc. = 5 × 10^−6^ M; 20 min; 25 °C; in darkness). The probe was excited at 366 nm (Ex slit = 2 nm), and fluorescence spectra were recorded at 400–550 nm (Em slit = 2nm) using a fluorescence spectrophotometer equipped with a xenon lamp (FS5 Spectrofluorometer; manufacturer: Edinburgh Instruments, Livingston, Scotland, UK). For analysis, general polarization (GP) was calculated as follows: the difference in the sum of fluorescence intensities (IFs) from 425 to 450 nm and the sum from 475 to 525 nm, divided by the sum of IFs from 425 to 450 nm and from 475 to 525 nm.

### 4.11. Observations of CaCdr1-GFP Localization in PM

The *C. albicans* strains AsCa1 (same as CAF2-1 but with Cdr1p-GFP) and KS023 (same as KS028 but with Cdr1p-GFP) were cultured for 8 h with starting OD_600_ = 0.1 (20 mL YPD, 28 °C, and 120 rpm) with or without supplementation with eugenol, extract 1, or extract 2 in ½xMIC50, MIC50, or 2xMIC50 concentrations. Then, the cells were harvested (4500 rpm, 5 min), washed twice with 0.9% saline solution, and concentrated. Microscopic observations were performed using a Zeiss Axio Imager A2 microscope with a Zeiss Axiocam 503 mono microscope camera and a Zeiss HBO100 mercury lamp (Poznań, Poland).

### 4.12. Statistical Analysis

Statistical analysis for growth, ROS level, PM permeabilization, and fluidity analysis was performed using a one-way ANOVA test (for different conditions inside one tested strain). To compare differences between strains in different conditions, the two-way ANOVA was applied. For the DPPH free-radical scavenging study, the *t*-test was used (binomial, unpaired). The data represents mean of 3 independent repetitions (if not indicated otherwise) ± SD, and significance was noted as follows: * *p* < 0.05; ** *p* < 0.01; *** *p* < 0.001.

## 5. Conclusions

**Figure 10:**
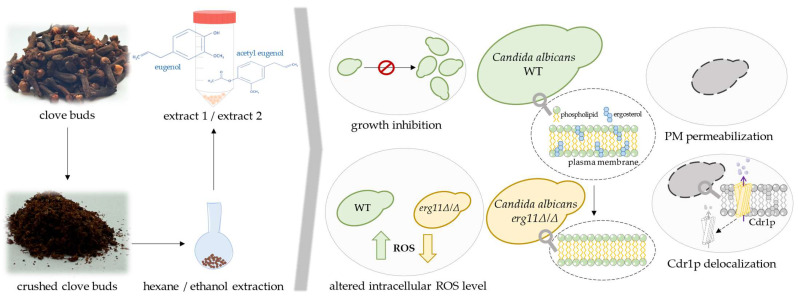
Graphical summary of key results obtained during this research. Figure illustrate method for obtaining tested extracts 1 and 2 (**left panel**) and influence of eugenol and extracts on C. albicans growth, ROS level, and plasma membrane (PM) integrity (**right panel**). Graphical summary was self-made and prepared using PowerPoint programme.

The general conclusions for the present study are illustrated in Figure 10. Extracts prepared from dried and crushed clove buds exhibited promising fungistatic properties against *C. albicans*. Yeast growth inhibition was observed for the same concentration of eugenol and extracts (%*v*/*v*), though the extracts at the used concentration contain less eugenol (µg/mL) than in a pure eugenol solution. Therefore, we conclude that those extracts contain other compounds that act synergistically with eugenol on *C. albicans* growth inhibition. An interesting observation is that eugenol and extracts lead to increased ROS levels in the *C. albicans* CAF2-1 (WT) strain, while in strains lacking ergosterol (KS028; *erg11Δ/Δ*), the initial ROS level is very high, and treatment with eugenol or extracts results in ROS reduction. Additionally, eugenol and extracts exhibit destructive activity against fungal PM, causing permeabilization and delocalization of PM-incorporated Cdr1p efflux pump. These findings are promising in context of potential clinical usage, considering the natural origin of extracts.

## Figures and Tables

**Table 1:** Eugenol content [mg/mL] in the tested extracts 1 and 2. Quantification of eugenol content in the extracts was calculated based on the standard curve for the eugenol standard (Supplementary Materials Figure S2).

	Extract 1	Extract 2
eugenol [mg/mL]	662.706	553.178

**Table 2:** Antioxidant properties of trolox (positive control), H_2_O_2_ (negative control), eugenol, extracts 1 and 2, and ergosterol. The concentration of eugenol and extracts 1 and 2 was equal to 0.039% *v*/*v* and represents the ½xMIC50 or MIC50 values for CAF2-1 (WT) or KS028 (*erg11Δ/Δ*) strains, respectively. Ergosterol, trolox, and H_2_O_2_ concentrations were 400, 500 μg/mL, and 0.0125% *v*/*v*, respectively. Methanol was used as a background. The tested compounds were incubated with DPPH for 90 min (37 °C, 200 rpm) and the absorbance at λ = 517 nm was recorded at specific time points: 0, 10, 60, and 90 min. The ability of scavenging the DPPH free radical was expressed in the decrease in A_517_, and % of DPPH inhibition was calculated according to the control (DPPH alone). Measurements were performed in 3 independent repetitions (±SD). Data for all the tested compounds were compared to eugenol at certain time points (* *p* < 0.05; ** *p* < 0.01; *** *p* < 0.001).

	t = 0 [min]	t = 10 [min]	t = 60 [min]	t = 90 [min]
trolox [500 μg/mL]	97.46 ± 0.29 ***	97.27 ± 0.29	97.01 ± 0.31	96.92 ± 0.32
H_2_O_2_ [0.0125% *v*/*v*]	15.38 ± 0.00 ***	14.21 ± 0.00 ***	10.96 ± 0.00 ***	8.79 ± 0.00 ***
eugenol [0.039% *v*/*v*]	79.22 ± 0.27	95.80 ± 0.54	96.13 ± 0.69	96.14 ± 0.64
extract 1 [0.039% *v*/*v*]	22.96 ± 1.73 ***	94.41 ± 0.39 *	95.61 ± 0.55	95.60 ± 0.56
extract 2 [0.039% *v*/*v*]	21.18 ± 0.57 ***	86.89 ± 0.17 ***	95.31 ± 0.40	95.22 ± 0.33
ergosterol [400 μg/mL]	3.23 ± 2.40 ***	10.46 ± 4.33 ***	32.55 ± 7.42 **	39.14 ± 8.75 **

**Table 3:** Percent (%) of *C. albicans* CAF2-1 (WT) and KS028 (*erg11Δ/Δ*) plasma membrane permeabilization after eugenol, extract 1, extract 2, and H_2_O_2_ treatment (concentration at MIC50 values corresponds with 0.078, 0.078, 0.078, and 0.0125% *v*/*v* for CAF2-1 and 0.0195, 0.039, 0.039, and 0.0063% *v*/*v* for KS028) was calculated according to the following formula: (number of total cells/number of PI-stained cells) × 100%. The experiment was performed in 3 biological repetitions, and data were compared to a control within *C. albicans* strain or between strains in certain conditions as indicated in the table (±SD; ns ≥ 0.05; ** *p* < 0.01; *** *p* < 0.001).

	Concentration	CAF2-1 [%]	KS028 [%]	Significance (CAF2-1 vs. KS028)
control	-	6.70 ± 0.74	15.07 ± 1.74 ***	ns
eugenol	½xMIC50	8.79 ± 1.00	57.61 ± 2.69 ***	***
	MIC50	9.76 ±1.32	55.51 ± 2.32 ***	***
	2xMIC50	82.23 ± 3.94 ***	58.24 ± 1.45 ***	***
extract 1	½xMIC50	41.13 ± 1.11 ***	87.46 ± 2.34 ***	***
	MIC50	45.67 ± 2.83 ***	82.89 ± 3.55 ***	***
	2xMIC50	90.98 ± 1.96 ***	88.26 ± 2.26 ***	ns
extract 2	½xMIC50	35.32 ± 1.57 ***	70.96 ± 2.23 ***	***
	MIC50	35.67 ± 1.40 ***	67.28 ± 3.05 ***	***
	2xMIC50	78.83 ± 5.82 ***	68.01 ± 2.39 ***	**
H_2_O_2_	½xMIC50	28.32 ± 4.01 ***	25.84 ± 1.59 **	ns
	MIC50	15.30 ± 3.22	69.54 ± 2.38 ***	***
	2xMIC50	30.96 ± 4.33 ***	82.35 ± 1.12 ***	***

**Table 4:** General polarization (GP) values determined for *C. albicans* CAF2-1 (WT) and KS028 (*erg11Δ/Δ*) after 4 h of treatment with eugenol or extract 1 or 2. MIC50 values were, respectively, CAF2-1—0.078, 0.078, 0.078, and 0.0125% *v*/*v*; KS028—0.0195, 0.039, 0.039, and 0.0063% *v*/*v*. Control was *C. albicans* cells cultured in YPD medium alone. The GP value was determined based on 6 independent, biological repetitions (±SD; * *p* < 0.05; ** *p* < 0.01; *** *p* < 0.001).

	Concentration	CAF2-1	KS028
control	-	−0.338 ± 0.028	−0.278 ± 0.029
eugenol	¼xMIC50	−0.240 ± 0.040 ***	−0.236 ± 0.032 *
	½xMIC50	−0.259 ± 0.052 *	−0.265 ± 0.043
	MIC50	−0.324 ± 0.008	−0.301 ± 0.043
extract 1	¼xMIC50	−0.253 ± 0.015 ***	−0.251 ± 0.019 *
	½xMIC50	−0.264 ± 0.016 ***	−0.273 ± 0.034
	MIC50	−0.290 ± 0.024 **	−0.308 ± 0.017 *
extract 2	¼xMIC50	−0.242 ± 0.026 ***	−0.232 ± 0.043 *
	½xMIC50	−0.256 ± 0.033 ***	−0.245 ± 0.034
	MIC50	−0.281 ± 0.013 ***	−0.279 ± 0.022
H_2_O_2_	¼xMIC50	−0.295 ± 0.022 **	−0.301 ± 0.006 *
	½xMIC50	−0.245 ± 0.070 *	−0.272 ± 0.021
	MIC50	−0.194 ± 0.047 ***	−0.240 ± 0.041

**Table 5:** The separation method for HPLC-UV analysis of extracts used in this study.

Time	Water + 0.1% HCOOH (%)	MeCN + 0.1% HCOOH (%)
Initial	75	25
1.0	75	25
5.0	5	95
7.0	5	95
7.1	75	25

**Table 6:** *Candida albicans* strains used in this study.

*C. albicans* Strain	Genotype	Source
CAF2-1	*ura3Δ::imm434/URA3*; wild-type	[32]
AsCa1	*ura3Δ::imm434/ura3Δ::imm434* *CDR1/CDR1-yEGFP-URA3*	[33]
KS023	*ura3Δ::imm434/ura3Δ::imm434* *CDR1/CDR1-yEGFP-URA3* *erg11Δ::SAT1-FLIP/erg11Δ::FRT* (parental strain: AscCa1)	[25]
KS028	*ura3Δ::imm434/URA3* *erg11Δ::SAT1-FLIP/erg11Δ::FRT* (parental strain: CAF2-1)	[25]

## Data Availability

Data is contained within the article and Supplementary Materials.

## Supplementary Materials

The following supporting information can be downloaded at https://www.mdpi.com/article/10.3390/ijms26178571/s1.
