# Supplementary material for: Multifunctional Activity of Syzygium aromaticum Extracts Against Candida albicans: Free Radicals, Membrane Permeabilization and Cdr1p Localization
Source: Int J Mol Sci. 2025 Sep 3;26(17):8571. doi: 10.3390/ijms26178571 (PMC12429323; doi:10.3390/ijms26178571)
Supplement: Supplementary file 1 [file ijms-26-08571-s001.zip › ijms-3736872-supplementary.pdf]

Supplementary Materials

S1. HPLC-UV analysis of composition of eugenol extracts

Chromatograms for different concentrations of eugenol standards are presented in Figure S1.

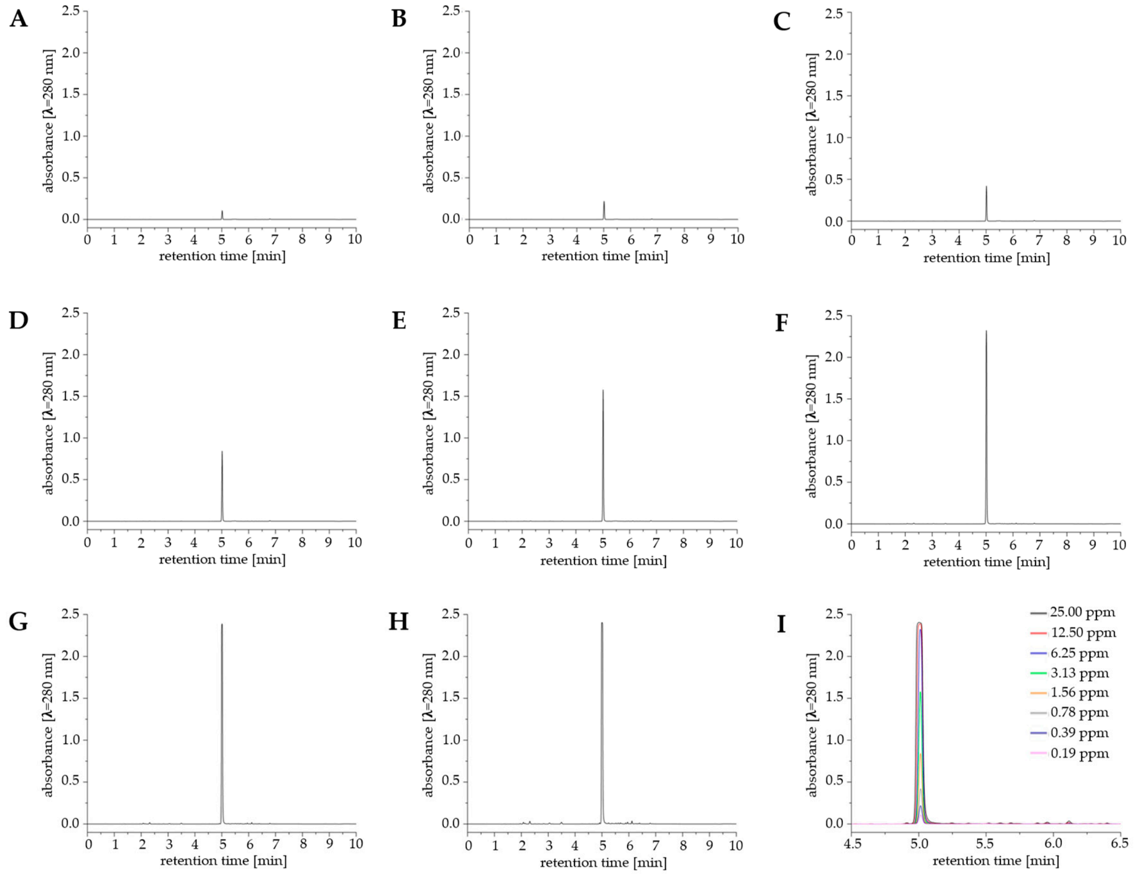

Figure S1. Chromatograms obtained after HPLC-UV analysis of eugenol standard (0.19 - 25 ppm where 1 ppm equals to 1 mg/mL; A - H). Merged chromatograms of different concentrations of eugenol are presented in Figure 1I. The detection was conducted using UV lamp set to λ = 280 nm.

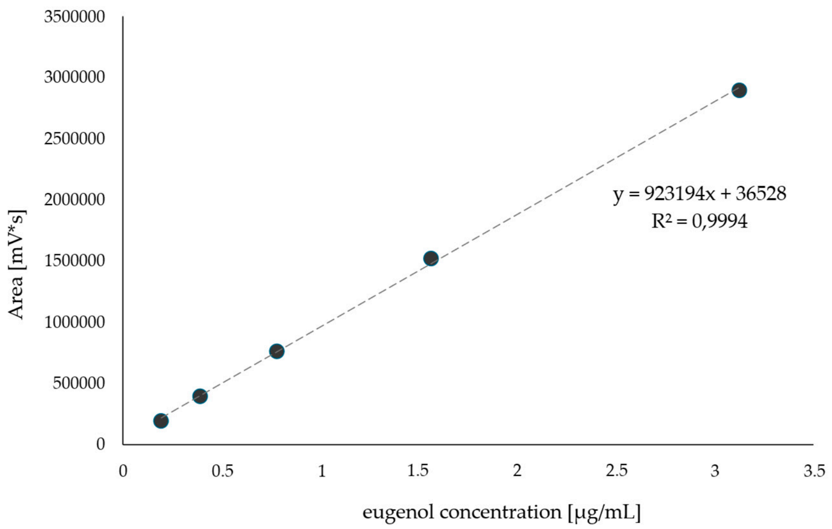

Figure S2. Standard curve for eugenol based on HPLC-UV analysis. Standard curve was then used to calculate the concentration of eugenol in tested extracts 1 and 2.

S2. Antifungal activity of eugenol extracts against Candida albicans

The antifungal activity of eugenol extract and H_2O_2 for AsCa1 (same as CAF2-1 but *CDR1*-GFP) and KS023 (same as KS028 but *CDR1*-GFP) is presented in Figure S3.

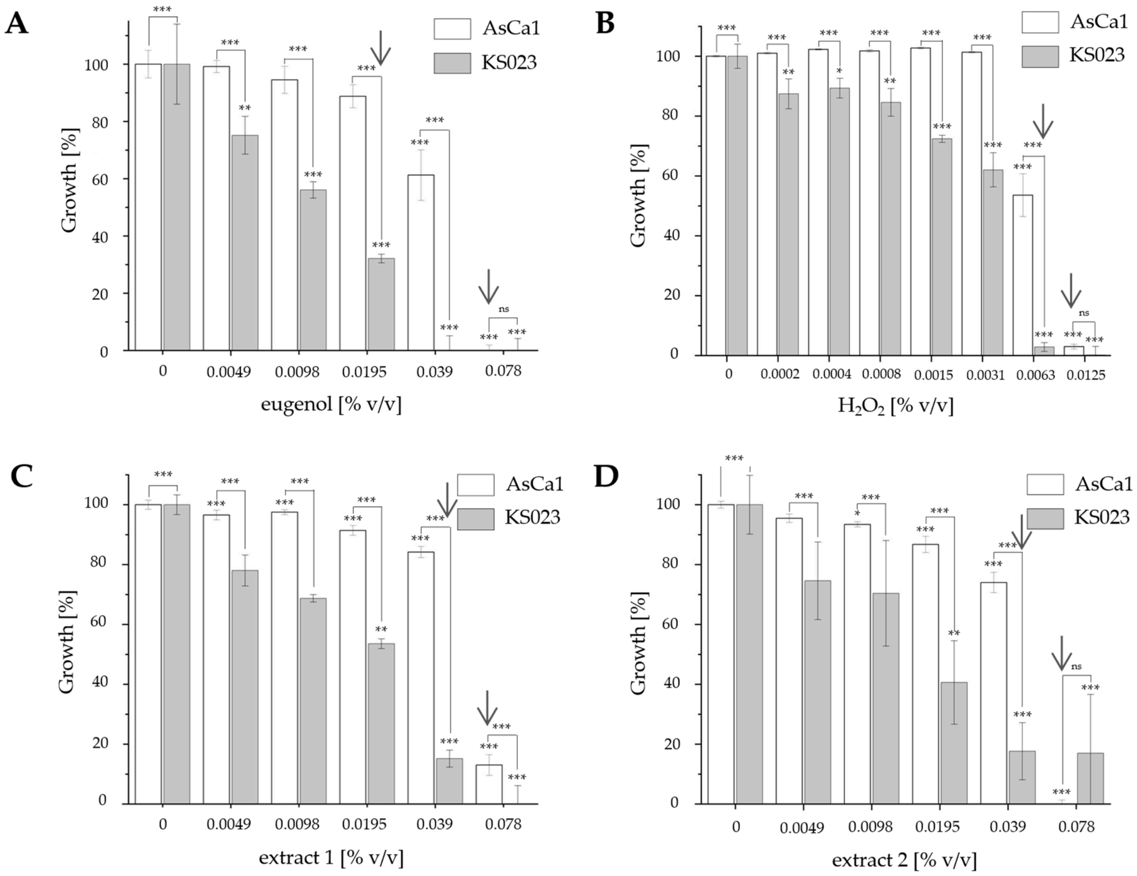

Figure S3. Growth of *C. albicans* AsCa1 (WT; Cdr1p-GFP) and KS023 (*erg11Δ/Δ*; Cdr1p-GFP) in presence of eugenol (**A**), extract 1 (**B**), extract 2 (**C**) and hydrogen peroxide (H_2O_2; **D**). *C. albicans* AsCa1 and KS023 were cultured in YPD medium supplemented (or not) with tested compounds on 96-well plates for 24 hours (28 °C, stationary). Then the optical density (OD) at λ = 600 nm was measured and the growth (%) was calculated comparing to OD_{600} for *C. albicans* cultured without presence of any compound (control conditions). Grey arrows indicate MIC50 for tested *C. albicans* strains. Experiment was performed in 3 biological repetitions (±SD; *, *p<0.05*; **, *p<0.01*; ***, *p<0.001*).

S3. Polyphenols content in tested extracts.

S3.1. Methodology for polyphenols content in tested extracts.

The Folin-Ciocâlteu method was applied to analyze the polyphenol content in tested extracts. 6 mL of ultra-pure H_2O_{dd} was mixed with 0.5 mL Folin-Ciocâlteu reagent (Merck, Darmstadt, Germany) and 0.1 mL of tested compounds. For standard curve preparation tested compounds solutions were replaced by 0.1 mL gallic acid (Merck, Darmstadt, Germany) solution (standard curve points were: 0.5; 0.4; 0.3; 0.2 and 0.1 mg/mL of gallic acid solutions). After 3 minutes of incubation the saturated sodium carbonate solution (1.5 mL) was added, and the solutions were subjected to 10 mL with ultra-pure H_2O_{dd}. Samples were then incubated in temperature 40 °C for 30 minutes and then absorbance at λ=765 nm was immediately analyzed using spectrophotometer Jenway 7205 (Cole-Parmer, Illinois, USA).

S3.2. Results of polyphenols content in tested extracts.

Results of polyphenols content analysis was presented in Figure S4.

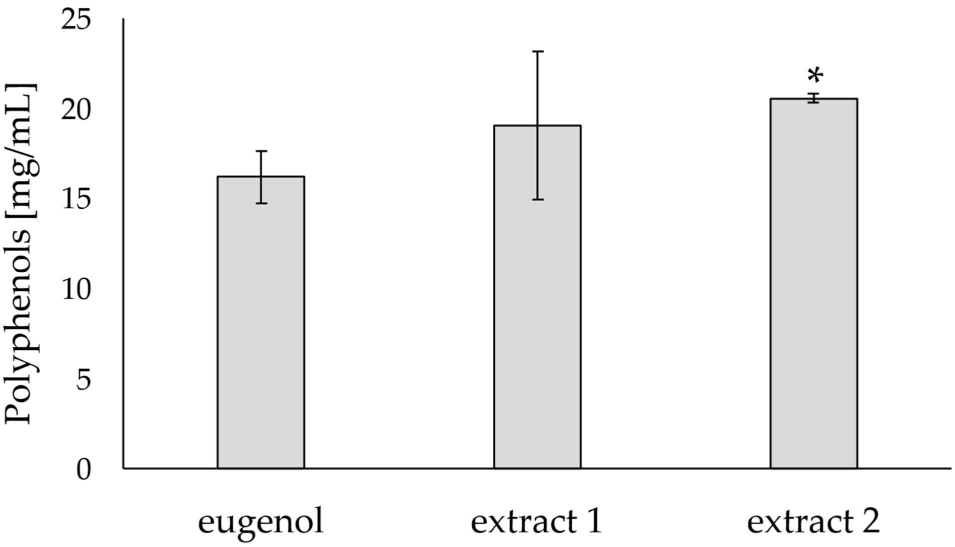

Figure S4. Polyphenols content [mg/mL] in eugenol, extract 1 and 2 detected by Folin-Ciocâlteu method. Results were compared to eugenol control using T-test (binomial, unpaired; *, *p<0.05*).

S4. Ergosterol determination after treatment with tested extracts.

S4.1. Methodology for determination of ergosterol content.

Ergosterol determination was performed based on Prajapati et al. protocol [1]. *C. albicans* were cultured for 24 hours in 20 mL of YPD medium (28 °C, 120 rpm) with or without (negative control): fluconazole (MIC50 concentration was 2 μg/mL), MIC50 concentration of eugenol, extract 1 and 2. Total sterols were extracted from harvested (4500 rpm, 10 minutes) and rinsed (sterile H_2O_{dd}) yeast cells (net wet mass was recorded for calculations) by addition of 3 mL of 25% KOH in methanol. Samples were incubated in temperature of 85 °C for 1 hour. After cooling down of samples, the 3 mL of n-hexane and H_2O_{dd} mixture (3:1 ratio) was added, vortexed for 3 minutes and allowed for phase separation. N-hexane layer was transferred to new glass vial and incubated in -20 °C for 24 hours.
After this time, samples were diluted 2-times with 100% ethanol and scanned in range of λ= 230 to 350 nm using spectrophotometer Jenway 7205 (Cole-Parmer, Illinois, USA). Blank was the n-hexane diluted identically as recorded samples.
The ergosterol reduction was calculated according to formulas:

$$\%\, ergosterol = \frac{F}{\text{cell mass [g]}}\left[\left(\frac{A_{282}}{290}\right) - \left(\frac{A_{230}}{518}\right)\right]$$

and then, to achieve % of ergosterol reduction:

$$ergosterol\ reduction\ (\%) = 100\% - \left(\frac{\text{investigated sample} \times 100\%}{\text{control sample (CAF2} - 1 \text{ negative control)}}\right)$$

The % of ergosterol reduction for tested samples were calculated in reference to *C. albicans* CAF2-1 negative control (cultured in YPD medium alone).

S4.2. Result of determination of ergosterol content.

Analysis of ergosterol reduction was provided in Table S1.

Table S1. Ergosterol reduction [%] after treatment of *C. albicans* CAF2-1 (WT) strain with FLC (2 μg/mL, positive control), ½MIC50 and MIC50 concentrations of eugenol, extract 1 and 2 or for *C. albicans* KS028 (*erg11Δ/Δ*) in control conditions (cultured in YPD medium alone) in accordance to *C. albicans* CAF2-1 negative control (cultured in YPD medium alone).

conditions	ergosterol reduction [%]
CAF2-1 (positive control; FLC)	100.00
KS028 (control conditions)	100.00
CAF2-1 (MIC50 eugenol)	84.57
CAF2-1 (MIC50 extract 1)	55.35
CAF2-1 (MIC50 extract 2)	91.07

References

1. Prajapati, J.; Goswami, D.; Dabhi, M.; Acharya, D.; Rawal, R.M. Potential dual inhibition of SE and CYP51 by eugenol conferring inhibition of Candida albicans: Computationally curated study with experimental validation. *Computers in Biology and Medicine*, **2022**, 151(A), 106237.
